# Fever of Unknown Origin and Atrial Fibrillation: A Case Report

**DOI:** 10.7759/cureus.32472

**Published:** 2022-12-13

**Authors:** Brandon W Knopp, Jeniel Parmar

**Affiliations:** 1 Internal Medicine, Florida Atlantic University Charles E. Schmidt College of Medicine, Boca Raton, USA; 2 Emergency Medicine, Florida Atlantic University Charles E. Schmidt College of Medicine, Boca Raton, USA

**Keywords:** non-valvular atrial fibrillation, infectious disease medicine, evidence-based management, case report, fever of unknown origin

## Abstract

Fever of unknown origin describes a temperature greater than 100.9°F which is present on multiple instances for a period over three weeks with no confirmed diagnosis despite a minimum of three outpatient visits, three days of inpatient testing, or one week of extensive outpatient testing. This diagnosis presents challenges in clinical management due to the unknown etiology. This case highlights a fever of unknown origin presenting with new-onset atrial fibrillation in a patient with no previous cardiac history. A 62-year-old Caucasian male presented to the ED with a nine-day history of intermittent fevers and chills. He returned from a rafting trip in North Carolina two weeks ago but reported no tick bites, animal encounters, or river water ingestion. Further evaluation was significant for an elevated white blood cell count and elevated inflammatory markers. Laboratory and radiologic testing for a wide array of infectious and malignant etiologies were unremarkable. Soon after hospital presentation, he developed a fever of 102.9°F with new onset palpitations and chest tightness due to atrial fibrillation. Episodes of atrial fibrillation continued for his seven-day hospital course with more severe symptoms in the evenings. He was administered broad-spectrum antibiotics and tested extensively with no definitive etiology. His fever curve downtrended with max temperatures below 100.9°F on hospital days six and seven with asymptomatic episodes of atrial fibrillation, prompting discharge. He continued to have low-grade fevers measured below 100.9°F for several days post-discharge with no associated symptoms, resulting in a diagnosis of fever of unknown origin following the 21st day. Fever of unknown origin is a clinical challenge, particularly in cases with no diagnosis discovered and cases with potentially life-threatening complications such as atrial fibrillation. This patient had multiple potential etiologies for his condition, but none had sufficient evidence for diagnosis, resulting in uncertainty regarding the ideal management. As a result, constant monitoring with supportive treatments and broad-spectrum antibiotics was utilized. These measures allowed for symptom remission and hospital discharge for outpatient follow-up. This case highlights a rare presentation of fever of unknown origin with new-onset atrial fibrillation in an otherwise healthy adult.

## Introduction

Fever is a common presenting complaint with many potential etiologies including infection, inflammatory conditions, malignancy, endocrine conditions, drug-induced, and idiopathic causes [1]. When a temperature greater than 100.9°F is present on multiple instances during a span of at least three weeks with no confirmed diagnosis despite appropriate clinical evaluation and diagnostic testing, it is classified as a fever of unknown origin. This diagnosis requires a comprehensive, yet reasonable investigation into fever etiology during a minimum of three outpatient visits, three days of inpatient testing, or one week of extensive outpatient testing [2]. This time requirement exists to allow sufficient time for appropriate investigations to be conducted including blood smears, blood cultures, urine cultures, viral cultures, imaging studies, and other pertinent testing [3].

Fever of unknown origin poses challenges in diagnosis and clinical management. As there is no initial diagnosis made following a comprehensive history, physical examination, and laboratory studies, management is largely supportive and focused on preventing complications until a definitive etiology is found [1,4]. This may include broad-spectrum antibiotics, antipyretics as needed, cessation of potentially causative medications, and hemodynamic optimization. Likewise, frequent reexamination and clinical assessment can help guide and direct care recommendations. As over 200 etiologies for FUO have been described, some cases go without a diagnosis despite appropriate diagnostic workup [1,5]. New-onset atrial fibrillation is a particularly concerning complication of FUO which can be triggered by fever and inflammation alone or secondary to the underlying cause of the FUO [6]. Only one case of FUO with new-onset atrial fibrillation has been reported to date, with the etiology found to be a primary cardiac lymphoma [7].

This case describes a rare presentation of fever of unknown origin with new-onset atrial fibrillation and no definitive fever etiology. The patient had multiple potential etiologies for his fever, but extensive history taking, physical examination, imaging, and laboratory studies were unremarkable for a definitive diagnosis. This study highlights a case of new-onset atrial fibrillation and a fever of unknown origin in an otherwise healthy patient, as well as the preventative measures taken to mitigate complications.

## Case presentation

A 62-year-old Caucasian male presented to the emergency department (ED) with a nine-day history of intermittent fevers and chills. Episodes occurred one to three times daily and were increasing in frequency and severity. Fevers developed at different times throughout the day with no identifiable triggers or exacerbating factors. He also experienced night sweats and headaches over the past nine days and fatigue over the past two months. He reported no sick contacts but had two recent procedures with an endovenous laser varicose vein surgery on his left leg six days ago and an incision and drainage of an abscess on his back 14 days ago. No antibiotics were prescribed following either procedure. He returned from a rafting trip in North Carolina two weeks ago as well where he went tubing in a river but did not go hiking. No tick bites, animal encounters, or river water ingestion were reported on further questioning.

**Figure 1:**
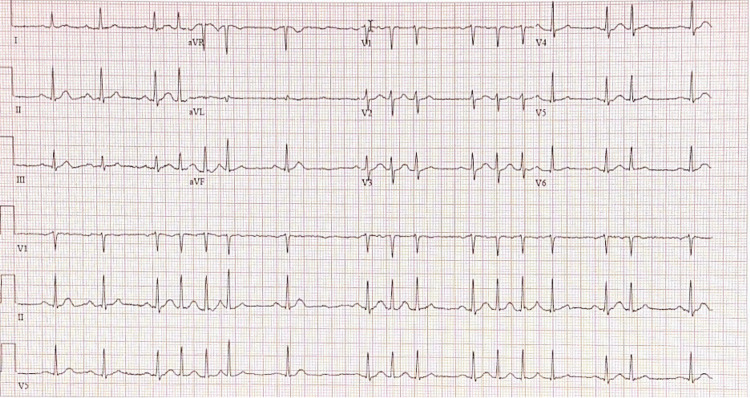
ECG on emergency department admission. Normal sinus rhythm with frequent and consecutive premature atrial complexes.

Vitals on admission were unremarkable. The physical examination was significant for left upper back erythema where the abscess drainage had occurred 14 days ago and 1+ ankle edema bilaterally. His initial laboratory results found an elevated alanine transaminase (ALT) (107 U/L), aspartate transferase (AST) (119 U/L), and a white blood cell count of 13.93 k/uL. He also had elevated inflammatory markers with a lactose dehydrogenase of 481 U/L, c-reactive protein (CRP) of 5.6 mg/dL, and procalcitonin of 0.18 ng/mL. Complete blood count (CBC) and complete metabolic panel (CMP) were within normal limits. A panel of rheumatologic and neoplastic markers was done with no positive results. He tested negative for a variety of infectious disease markers (Table 1). Three separate blood cultures, one taken three days before ED presentation at the ED of a nearby medical center and two taken on admission, had no growth after five days. The blood smear was unremarkable. ECG showed normal sinus rhythm with frequent and consecutive premature atrial complexes (Figure 1).

**Table 1:** Infectious disease panel. EBV: Epstein-Barr virus; VCA: viral capsid antigen

Marker	Result
Hepatitis A antibody, IgM	Negative
Hepatitis B core antibody, IgM	Negative
Hepatitis B surface antigen	Negative
Hepatitis C antibody	Negative
Mononucleosis slide test	Negative
Lyme IgG/IgM	Negative
Babesia microti IgG	Negative
Rocky Mountain spotted fever IgG/IgM	Negative
Typhus fever IgG/IgM	Negative
HIV 1/2 Combo (antigen/antibody)	Negative
Leptospira IgM antibody	Negative
EBV VCA IgM	Negative
Toxoplasmosis IgM	Negative
Adenovirus	Negative
Coronavirus	Negative
Influenza virus A/B	Negative
Parainfluenza virus 1-4	Negative
Human metapneumovirus	Negative
Mycoplasma pneumoniae	Negative
Cryptosporidium	Negative
Malaria screen	Negative

An x-ray revealed no acute cardiopulmonary process but a pan CT scan (head, neck, chest, abdomen, and pelvis) done to evaluate for solid masses or abscesses showed a 7 mm nodule favored to be a lymph node in the right upper lung lobe. The nodule was unchanged from a CT scan six months prior. Given the unchanged size and risks of complications, no attempt at diagnostic biopsy was made. Right upper quadrant ultrasound evaluated the liver in the setting of elevated transaminases and found non-specific hepatocellular changes potentially representing fatty infiltration. A transthoracic echocardiogram (TTE) found an ejection fraction of 55 with no valvular or contractile abnormalities, though the study was of poor quality due to obese body habitus. Urinalysis results were likewise insignificant and lower extremities Dopplers found no evidence of deep venous thrombosis.

**Figure 2:**
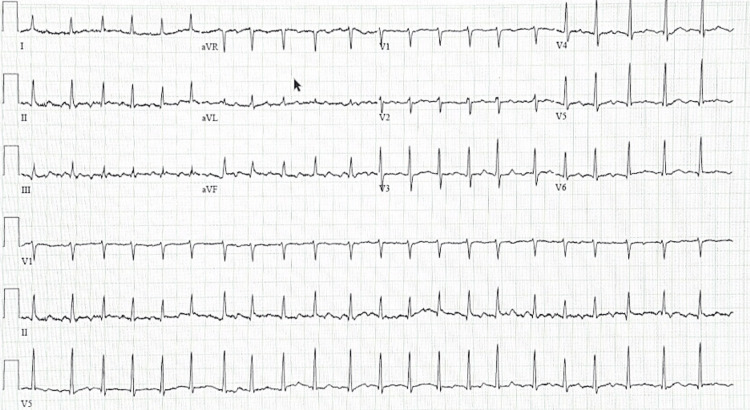
ECG 75 minutes post-ED admission. Atrial fibrillation with rapid ventricular response.

Approximately 75 minutes after the hospital presentation, he developed a fever of 102.9°F and reported new onset palpitations and chest tightness with the ECG showing atrial fibrillation with a rapid ventricular response of up to 160 beats per minute (Figure 2). His symptoms improved with a 1 L bolus of lactated Ringer’s solution and resolved within 30 minutes. He experienced similar episodes of atrial fibrillation with a rapid ventricular response each day of his seven-day hospitalization with more severe symptoms in the evenings. The severity of his symptoms peaked during the third night of his hospitalization with moderate chest tightness, shortness of breath, palpitations, and a pulse in the 150s lasting around 30 minutes.

**Figure 3:**
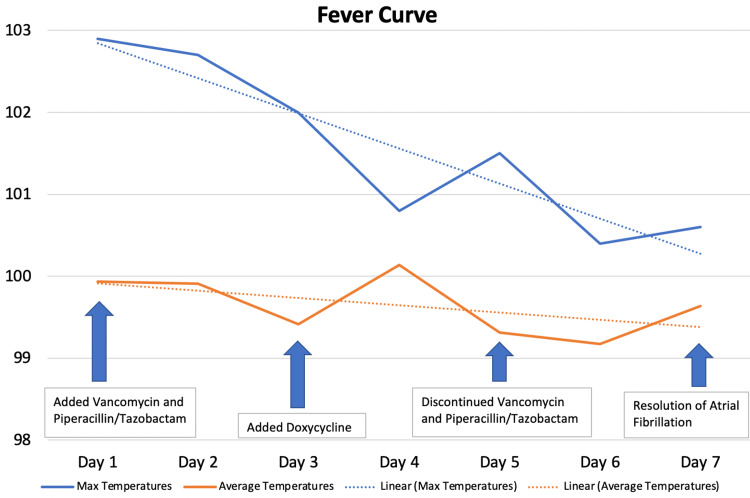
Fever curve during hospitalization.

In the absence of evidence supporting a top diagnosis, the patient was admitted with telemetry monitoring. He was administered vancomycin and piperacillin/tazobactam on admission, which were later discontinued on day five with doxycycline added on day three. His fever curve downtrended each day with a max fever peak of 102.9°F on hospital day one (Figure 3). Likewise, his symptoms decreased in severity with continued episodes of atrial fibrillation with a rapid ventricular response which were asymptomatic on the last two days of his hospital stay. He was discharged with a two-week course of doxycycline and scheduled for outpatient follow-up with no confirmed etiology for his symptoms. Low-grade fevers continued for several days following discharge with no associated symptoms and, as a result, he was diagnosed with a fever of unknown origin. One week later, outpatient TTE was unremarkable with no significant findings. Ambulatory cardiac monitoring which began one week after hospital discharge noted normal sinus rhythm with no episodes of atrial fibrillation in the following month.

## Discussion

The large number of potential differential diagnoses makes diagnosing the etiology of a fever of unknown origin particularly challenging. Our patient had no major risk factors or family history of a condition that may have caused his fever and atrial fibrillation. His fevers were intermittent for around three weeks with one week of recorded episodes of atrial fibrillation. This pattern of symptoms with no additional signs or symptoms to suggest an etiology makes this case unique, particularly as extensive laboratory and imaging studies were unremarkable. His recent travel to North Carolina was concerning for a potential tick-borne pathogen, his recent operations with abscess drainage were potentially causative of bacteremia and the enlarged lymph node in his right upper lobe with "B" symptoms made leukemia and lymphoma potential etiologies as well.

Each of these concerns, as well as less likely causative conditions, were lowered on the differential diagnosis following subsequent laboratory and imaging studies. Hematological studies including three separate blood cultures and blood smears were unremarkable. Extensive history revealed no additional risk factors related to animal exposure, unpasteurized dairy consumption, or symptoms characteristic of Coxiella or Brucella infection to warrant additional testing for infectious etiologies. Neoplastic etiologies capable of causing fever including leukemia, lymphoma, hepatocellular carcinoma, and renal cell carcinoma were considered as well [8]. However, the patient had no weight loss, organomegaly, anemia, thrombocytopenia, lymphadenopathy, or other features suggestive of neoplasm on clinical examination and laboratory studies. Pan CT scan also found no solid masses and an enlarged pulmonary lymph node was unchanged from imaging six months ago. The patient tested negative for a panel of rheumatologic factors, had no family history of rheumatologic disease, and had few symptoms suggestive of rheumatologic disease, decreasing the likelihood of a rheumatologic etiology. In evaluating his atrial fibrillation, both TTE and chest x-ray were unremarkable for acute processes. He had hypertension treated with nebivolol and furosemide, but no personal or family history of arrhythmias or other cardiac conditions, aside from his father who had "bad heart valves."

Testing was focused on potential etiologies for fever rather than broad testing for non-evidence-based investigations, as recommended to better target likely diagnoses and reduce costs [9]. While more invasive testing, such as lymph node biopsies, could have potentially yielded a diagnosis, diagnostic tests were only recommended if appropriately evidence-based. This approach avoided further patient discomfort and focused on recovery, as up to 75% of reported cases resolve spontaneously [10]. The downtrending fever curve and decreasing severity of episodes of atrial fibrillation showed the efficacy of this approach, even in the absence of a definitive diagnosis.

As there was a notable risk for complications, such as worsening arrhythmias and hemodynamic instability, the patient was carefully managed during his hospitalization. However, given the waning severity of symptoms and minimal discomfort before discharge, the patient was deemed stable for outpatient follow-up. He was discharged with a two-week course of doxycycline given the apparent response in temperatures, but no anticoagulation due to the absence of symptomatic atrial fibrillation episodes since hospital day six. Ambulatory cardiac monitoring which began one week after hospital discharge revealed no episodes of atrial fibrillation in the following month. An infectious etiology was presumed given the response to doxycycline and conversion to sinus rhythm following fever resolution. This case is atypical in that the fever of unknown origin was complicated by new-onset atrial fibrillation and no diagnosis was reached, complicating recommendations for outpatient care.

## Conclusions

Advanced imaging and diagnostics have decreased the prevalence of fever of unknown origin due to earlier detection, however, the etiology of some fever of unknown origin cases is never determined. This patient’s case was presumed to be due to an insect-born pathogen from rafting in North Carolina, though testing found no evidence of this etiology. Likewise, alternative etiologies including inflammatory, malignancy, endocrine, drug-induced, and idiopathic causes were evaluated. This case highlights a rare presentation of a fever of unknown origin with new-onset atrial fibrillation in an otherwise healthy adult and is an example of evidence-based care for a patient presenting with a fever of unknown origin.
